# High Glucose Activates YAP Signaling to Promote Vascular Inflammation

**DOI:** 10.3389/fphys.2021.665994

**Published:** 2021-06-04

**Authors:** Jeremy Ortillon, Jean-Christophe Le Bail, Elise Villard, Bertrand Léger, Bruno Poirier, Christine Girardot, Sandra Beeske, Laetitia Ledein, Véronique Blanchard, Patrice Brieu, Souâd Naimi, Philip Janiak, Etienne Guillot, Marco Meloni

**Affiliations:** ^1^Cardiovascular Research Unit, Sanofi R&D, Chilly-Mazarin, France; ^2^Molecular Histopathology and Bio-Imaging Translational Sciences, Sanofi R&D, Chilly-Mazarin, France

**Keywords:** YAP/TAZ, diabetes, inflammation, endothelial cells, vascular complications

## Abstract

**Background and Aims:**

The YAP/TAZ signaling is known to regulate endothelial activation and vascular inflammation in response to shear stress. Moreover, YAP/TAZ signaling plays a role in the progression of cancers and renal damage associated with diabetes. However, whether YAP/TAZ signaling is also implicated in diabetes-associated vascular complications is not known.

**Methods:**

The effect of high glucose on YAP/TAZ signaling was firstly evaluated *in vitro* on endothelial cells cultured under static conditions or subjected to shear stress (either laminar or oscillatory flow). The impact of diabetes on YAP/TAZ signaling was additionally assessed *in vivo* in *db/db* mice.

**Results:**

*In vitro*, we found that YAP was dephosphorylated/activated by high glucose in endothelial cells, thus leading to increased endothelial inflammation and monocyte attachment. Moreover, YAP was further activated when high glucose was combined to laminar flow conditions. YAP was also activated by oscillatory flow conditions but, in contrast, high glucose did not exert any additional effect. Interestingly, inhibition of YAP reduced endothelial inflammation and monocyte attachment. Finally, we found that YAP is also activated in the vascular wall of diabetic mice, where inflammatory markers are also increased.

**Conclusion:**

With the current study we demonstrated that YAP signaling is activated by high glucose in endothelial cells *in vitro* and in the vasculature of diabetic mice, and we pinpointed YAP as a regulator of high glucose-mediated endothelial inflammation and monocyte attachment. YAP inhibition may represent a potential therapeutic opportunity to improve diabetes-associated vascular complications.

## Introduction

High glucose-induced endothelial dysfunction is a crucial initiating factor in the development of diabetes-associated vascular complications which, in turn, are responsible for shortened life expectancy, high rate of hospitalization, and high morbidity and mortality in patients with diabetes (Grundy et al., 2002; Schalkwijk and Stehouwer, 2005; Beckman and Creager, 2016).

The diabetic endothelium is characterized by increased expression of adhesion molecules and proinflammatory cytokines, resulting in a prothrombotic and proinflammatory state that favors the development of atherosclerosis (Anderson et al., 1995; Plutzky, 2003). Recent studies based on gene deletion have demonstrated the implication of YAP (Yes-Associated Protein) and TAZ (Transcriptional coactivator with PDZ-binding motif) in regulating endothelial activation and vascular inflammation (Lv et al., 2018). YAP and TAZ are transcriptional regulators and the main downstream mediators of the Hippo pathway, which regulates cell proliferation, survival and differentiation, thus controlling organ development and tissue regeneration (Pan, 2010; Yu and Guan, 2013; Piccolo et al., 2014). When the Hippo pathway is active, YAP and TAZ are phosphorylated/inactivated resulting in cytoplasmic localization and subsequent ubiquitin-mediated degradation (Zhao et al., 2010). Inhibition of the Hippo pathway promotes YAP and TAZ dephosphorylation and activation, followed by their translocation into the nucleus where they interact with the transcriptional enhancer associated domain (TEAD) leading to the activation of target genes associated with cell proliferation and differentiation (Piccolo et al., 2014).

The YAP/TAZ signaling is implicated in the regulation of vascular mechanotransduction and homeostasis (Dupont et al., 2011), and participates in the early structural and inflammatory events that occur in response to shear stress variation in large arteries (Wang et al., 2012; Choi et al., 2018; He et al., 2018). Wang et al. (2012) have demonstrated that YAP expression increases after carotid artery injury, and that YAP/TAZ mediates vascular remodeling during the progression of carotid stenosis. Moreover, Xu and colleagues have shown that YAP/TAZ inactivation protects against endothelial inflammation in the setting of laminar flow (Xu et al., 2016). Furthermore, turbulent flow has been demonstrated to activate YAP/TAZ and promote an atheroprone phenotype characterized by increased endothelial cell dysfunction and inflammation in the mouse carotid artery (Wang K. C. et al., 2016). Conversely, laminar flow was shown to inhibit RhoA (which is known to regulate YAP signaling), thus leading to YAP phosphorylation/inactivation (Wang K. C. et al., 2016). In addition, a role for YAP/TAZ signaling has also been unveiled in the context of diabetes: in fact, it has been indicated that YAP favors cancer incidence and progression in patients with diabetes, and that it plays an important role in diabetes-associated renal damage (Chen and Harris, 2016; Wang C. et al., 2016; Zhang et al., 2018; Ma et al., 2019). It is not known, however, whether YAP/TAZ signaling is also implicated in diabetes-associated vascular complications.

With the current study we investigated the importance of YAP/TAZ signaling on endothelial cell activation and vascular inflammation in the settings of diabetes. Using *in vitro* and *in vivo* approaches, we demonstrated that YAP signaling is activated in the diabetic vasculature and plays a role in the regulation of endothelial inflammation and monocyte attachment induced by high glucose.

## Materials and Methods

### Cell Culture

Human umbilical vein endothelial cells (HUVECs, PromoCell) were cultured in endothelial cell medium (PromoCell) added with Growth Medium Supplement Pack (PromoCell) in a humidified atmosphere at 37°C and 5% CO_2_. Human aortic endothelial cells (TeloHAEC) were immortalized by stably expressing human telomerase catalytic subunit hTERT (ATCC). In addition, TeloHAECs were also stably transduced with the IncuCyte^TM^ NucLight^TM^ red Fluorescent Protein Lentivirus Reagents (Essen BioScience) and with Tead Luciferase lentiviral construct (Vectalys) containing wild type or 7× mutated (inactive form) TEAD-responsive synthetic element/region (8×GTIIC region followed by a minimal chicken TNNT2 promoter, before the luciferase gene, called TEADwt-Luc and TEADmut-Luc, respectively) driving luciferase expression (Kim and Gumbiner, 2015). To mimic the diabetes environment, HUVECs or TeloHAECs were cultured with high glucose (15 or 25 mM) for 24 h under static conditions. In order to replicate the *in vivo*-like flow conditions of an artery, HUVECs were additionally exposed to fluidic conditions. Briefly, HUVECs (2,5 × 10^5^ cells) were seeded onto glasses 0.4 mm Luer μ-slides I (Ibidi; channel length, 50 mm; channel width, 5 mm; and channel height, 0.4 mm) coated with rat tail collagen I (Gibco; 100 μg/ml in PBS) and grown until confluence. The slides were assembled into flow chambers and connected to an Ibidi Pump System (Ibidi). HUVECs were cultured with high glucose (25 mM) and exposed to steady laminar shear stress (12 dyn/cm^2^) or oscillatory shear stress (0.5 ± 6 dyn/cm^2^; 1 Hz) for 72 h without renewal of the culture medium. Glucose concentration at baseline (0 h) or after 72 h was not assessed: given the volume of medium used in the Ibidi Pump System (12 ml) and based on previous reports (Barcelos et al., 2009; Mangialardi et al., 2013; Zhang J. et al., 2019), glucose concentration is not expected to change in these experimental settings. The flow system was maintained at 37°C, and the circulating medium was equilibrated with a humidified atmosphere of 5% CO_2_. For all experiments, HUVECs were used between P2 and P5. Human monocytic cell line THP-1 (Leibniz Institute DSMZ-German Collection, GmbH) were transfected with the IncuCyte^TM^ NucLight^TM^ Green Fluorescent Protein (GFP) Lentivirus Reagents (Essen BioScience). THP-1-GFP were cultured in RPMI 1640 medium (Gibco) and 10% FBS (Gibco).

### *In vitro* YAP/TAZ Inhibition

Inhibition of the YAP/TAZ signaling pathway was obtained by using either siRNAs against YAP and TAZ or the small molecule TEAD inhibitor K-975 (Kaneda et al., 2020). For siRNA-mediated inhibition of YAP/TAZ, HUVECs were transfected with siRNAs using Lipofectamine^TM^ RNAiMAX reagent (Thermo Fischer) in Opti-MEM and L-Glutamine (Gibco) according to the manufacturer’s instruction. Endogenous YAP/TAZ expression was knocked down by transfecting cells with 100 nM of siRNA against YAP (Silencer-select siRNA s20366, Ambion) and TAZ (ON-TargetPlus siRNA, Horizon discovery). Control cells were transfected with negative control siRNA (Ambion). Cells were incubated with transfection complexes at 37°C in 5% CO2 for 4 h and media was replaced with complete endothelial growth medium for 20 h. Then, HUVEC were cultured for additional 24 h on either normal (5 mM) or high glucose (25 mM) condition in endothelial cell medium added with Growth Medium Supplement Pack (PromoCell). For the purpose of TEAD inhibition, K-975 was synthesized internally based on Kaneda et al. (2020). K-975 at 200 nM was added to HUVECs cultured on either normal (5 mM) or high glucose (25 mM) for 24 h, and under either static conditions or subjected to fluidic conditions. DMSO (0.01%, v/v) was used as control.

### Luciferase Assay

TeloHAECs were seeded at the density of 15 × 10^3^ cells/well in a 96-well plate format and maintained in a complete medium (CM) composed of Vascular Cell basal medium (BM), 5.5 mM glucose supplemented with Vascular Endothelial Cell Growth Kit-VEGF with 2% FBS and cytokines (ATCC), and incubated at 37°C in 5% CO_2_ for 24 h. Then, the medium was removed, and cells were kept in BM supplemented with 0.1% BSA with or without K-975, or under high glucose conditions (25 mM). After 24 h of incubation, the fluorescence intensity was detected by a fluorescence plate reader (Clariostar, BMG Labtech). Then, cells were lysed and the Luc-Screen^TM^ Extended-Glow Luciferase Reporter Gene Assay System (Invitrogen) was used to detect luciferase activity (according to the manufacturer’s instruction). This reporter assay allows a direct investigation of the interaction between YAP/TAZ and TEAD: if TEAD interacts with YAP/TAZ the luciferase activity increases; if their interaction is prevented (i.e., by a TEAD inhibitor like K-975) the luciferase activity decreases.

### Monocyte Adhesion Assay

The adhesion assay was performed by perfusing the THP-1-GFP positive cells on HUVECs cultured under either normal or high glucose conditions (with or without K-975, 200 nM), and already subjected to laminar flow (12 dyn/cm2) for 72 h. THP-1-GFP were resuspended at the concentration of 5 × 10^5^ cells per mL in RPMI 1640 (supplemented with 10% serum), then perfused on the HUVECs monolayer for 60 min at 37°C. To allow THP-1 cells attachment to HUVECs, the laminar flow conditions were set at 5 dyn/cm^2^. After removing the non-attached THP-1 cells (by washing with RPMI), fluorescent images were taken using an IN Cell Analyser 2200 (GE Healthcare). To distinguish THP-1-GFP cells from HUVECs, HUVECs were prelabelled with Hoechst (Invitrogen). IN Cell Analyser software was used to quantify the number of adherent THP-1 cells.

### Western Blot

Human umbilical vein endothelial cells were lysed in ice-cold cell extraction Buffer (Invitrogen) supplemented with protease/phosphatase inhibitor mixture (Sigma). Protein concentration was determined with the Pierce BCA Protein Assay Kit (Thermo Fischer). Equal amounts of protein (10 μg) were loaded and resolved by SDS/PAGE. After electrophoresis, proteins were transferred to PVDF membranes, followed by blocking with 10% blotting-Grade Blocker (Bio-Rad). The membranes were incubated with primary antibodies against phospho-YAP (Ser-127) (1:1,000), YAP (1:1,000), phospho-TAZ (Ser-89) (1:1,000), TAZ (1:2,500), VCAM-1 (1:1,000), ICAM-1 (1:1,000), cytokines connective tissue growth factor (CTGF; 1:1,000), cysteine-rich angiogenic inducer 61 (CYR61; 1:1,000) (all from Cell Signaling), GAPDH (Santa Cruz Biotechnologies, 1:10,000), β-actin (Sigma, 1:10,000) with gentle agitation overnight at 4°C. The membranes were washed three times for 10 min each with TBS-T (containing 0.1% Tween-20) and incubated with horseradish-peroxidase (HRP)-conjugated anti-mouse-IgG (Jackson ImmunoResearch, 1:10,000) or HRP-conjugated anti-rabbit-IgG (Jackson ImmunoResearch, 1:10,000) followed by chemiluminescence detection using GE Healthcare ECL western blotting substrate according to the manufacturer’s protocol. The blot image was acquired using G:BOX Chemi XL 1.4 (Syngene) imaging system and the band density was quantified by using GENETOOLS V4.03 (Syngene) software.

### qPCR

Total RNA was isolated from HUVECs by using the Maxwell RSC simply RNA Tissue kit (Promega). The concentration of RNA was determined by using a NanoDrop spectrophotometer (Thermo Fischer). One microgram of total RNA was reversely transcribed into cDNA by using SuperScript VILO cDNA Synthesis Kit (Invitrogen). The real-time PCR was carried out with TaqMan Universal PCR Master Mix (Applied Biosystems) on Stratagene Mx3000P (Agilent Technologies) detection system. Primer sets used in our study (predesigned by Thermo Fischer) were as follows: CTGF (Hs00170014_m1); CYR61 (Hs00155479_m1). The relative gene expression was determined by 2^–ΔΔCt^ method.

### Flow Cytometry Analysis

Human umbilical vein endothelial cells were briefly washed with sterile HBSS (Gibco), detached from the plate using versene with 5% of trypsin-EDTA (0.025%, v/v), then washed with staining buffer (BD Pharmingen). Cells were then transferred to a FACS plate, washed and resuspended in 10% PE-conjugated mouse anti-human ICAM-1 and FITC-conjugated mouse anti-human VCAM-1 (both from BD Pharmingen) in staining buffer for 30 min on ice and in the dark. Then cells were washed, resuspended in HBSS and analyzed using a Guava EasyCyte flow cytometer (Merck-Millipore). Data analysis was performed using the Guava software (Merck-Millipore). Mouse IgG1κ-FITC and PE were used as isotype controls.

### Animals and Ethics Statement

Diabetic male *db/db* mice and sex- and age-matched non-diabetic *db/* + control mice (on the C57BL/6J background; Jackson Laboratories) were used to conduct animal studies. Mice were housed with free access to standard chow diet (Harlan) and water. Blood glucose levels were measured before tissue collection with Accu-Chek^®^ glucose meter (Roche). All procedures involving animals were performed in agreement with the European Community standard on the care and use of laboratory animals (2010/63/UE) and were approved by the IACUC of Sanofi R&D. All procedures were performed in AAALAC-accredited facilities in full compliance with the recommendations of the French Ministry of Research.

### *In vivo* Alteration of Shear Stress

To induce changes in shear stress, a perivascular shear stress modifying cast was tied around the right common carotid artery of 18-week-old mice for 4 weeks. The cast consists of 2 longitudinal halves which, when put together, form a cylinder with an inner diameter of 500 μm (non-constrictive) that gradually declines to 250 μm, thus producing a gradual stenosis and gradual increase in shear stress (Cheng et al., 2005) (Sepran; Sepra, Delft, Netherlands). Briefly, mice were anesthetized with isoflurane, and the right anterior cervical triangle was accessed by incision. The right common carotid artery was dissected from connective tissue and both halves were placed and tied with a suture. Wounds were closed and animals were allowed to recover. The left carotid artery was left untouched and served as control.

### Immunohistochemical Analyses

Twenty-two-week-old mice were euthanized by exsanguination under profound anesthesia and immediately perfusion-fixed with heparinized PBS followed by 4% formalin. Both left (control) and right carotid arteries were removed and embedded in paraffin. All samples were cut into transverse sections (5 μm in thickness) and prepared for subsequent immunohistochemical analyses. For cast-instrumented arteries, sections were cut approximately 250 μm downstream of the cast, in the region of low and oscillatory shear stress (Cheng et al., 2005). Immunostaining were performed using the Ventana Discovery XT or BenschMark Ultra automated Systems (Ventana Medical System, Inc., Roche) with standard DabMab or UltraMab detection systems. Paraffin-embedded sections were incubated with primary antibodies against phospho-YAP (Ser127) (D9W2I, Cell Signaling, 1:1,000), YAP (D8H1X, Cell Signaling, 1:50) and VCAM-1 (ab134047, Abcam, 1:500). Optimal concentrations for each antibody were determined by individual pilot studies. Samples were counterstained with hematoxylin II and bluing reagent (Ventana) and coverslipped with Pertex. For all immunostaining, images of the entire slides were acquired on an Olympus Virtual Slide scanner (VS120) and quantitatively analyzed on whole tissue sections using Area Quantification Brightfield v1.0 module of Indica Labs HALO software (based on the intensity of the immunolabeling). Briefly, the intima-media area of the analyzed sections was manually delimited on images and positive stained area including weak (in yellow), moderate (in orange) and strong (in red) positive pixels was automatically quantified for each animal (as illustrated in Supplementary Figure 1).

### Statistical Analysis

Values are presented as mean ± standard error of mean (SEM). Non-parametric two-tailed Mann-Whitney test or parametric two-tailed Student’s *t* test was used for comparisons between two groups, and non-parametric Kruskal-Wallis test was used to compare more groups, respectively. Statistical analysis was performed using GraphPad Prism software 8.0.2 A *p* value < 0.05 was interpreted to denote statistical significance.

## Results

### High Glucose Induces YAP Activation in Endothelial Cells

**FIGURE 1:**
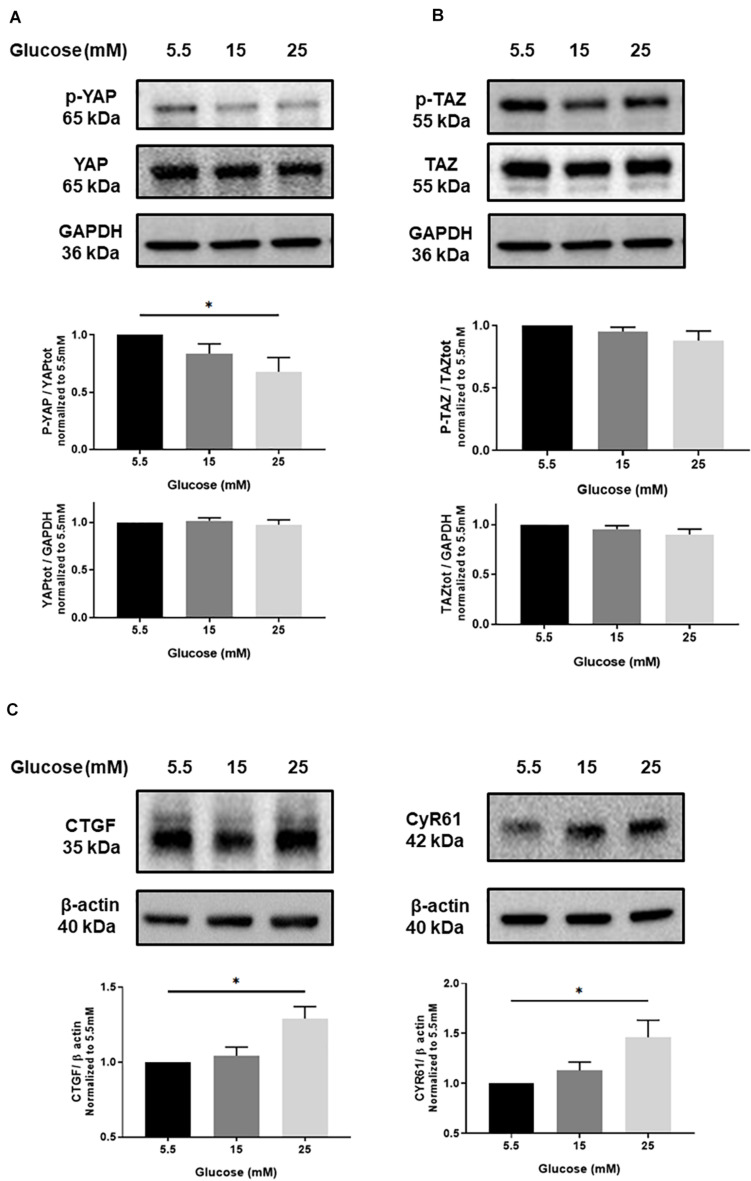
Effect of high glucose on YAP activation in endothelial cells. Representative protein expression blots and bar graphs of phospho-YAP (p-YAP; S127) and total YAP **(A)**, phospho-TAZ (p-TAZ; S89) and total TAZ **(B)**, CTGF and CYR61 **(C)** in HUVECs cultured under static conditions with different concentrations of glucose for 24 h. The control images of GAPDH are re-used for illustrative purposes. Data are presented as the mean ± SEM. *N* = 5/group for panels A and B; *N* = 7/group for panel C. *p < 0.05 between the indicated groups. Two-tailed Student’s *t* test was used for comparisons between the groups.

**FIGURE 2:**
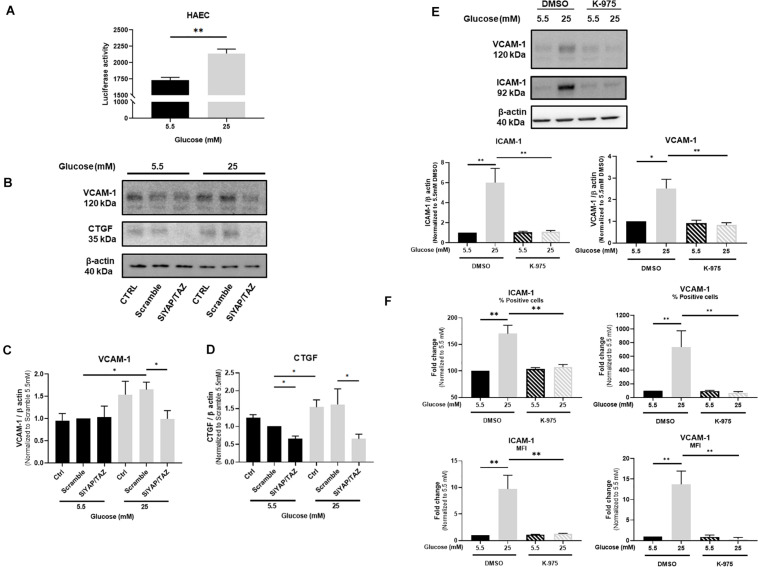
YAP mediates glucose-induced endothelial activation. Activity of TEAD-luciferase in TeloHAECs cultured under either normal or high glucose **(A)**. Representative protein expression blots **(B)** and bar graphs showing the expression of VCAM-1 **(C)** and CTGF **(D)** in HUVECs cultured under static conditions with either normal or high glucose and additionally treated with siRNA for YAP/TAZ. Representative protein expression blots and bar graphs showing the expression of VCAM-1 and ICAM-1 **(E)** as well as their percentage (%) and cell surface expression (represented as mean of fluorescence – MFI) assessed by flow cytometry **(F)** in HUVECs cultured under static conditions with either normal or high glucose for 24 h and in the presence, or not, of the YAP/TEAD inhibitor K-975 at 200 nM. DMSO was used as control. Data are presented as the mean ± SEM. *N* = 3/group for panel A; *N* = 4/group for panel C; *N* = 6/group for panel D; *N* = 6–7/group for panel E; *N* = 5/group for panel F. ^∗^*p* < 0.05 and ^∗∗^*p* < 0.01 between the indicated groups. Two-tailed Mann-Whitney test was used for comparisons between the groups.

A series of *in vitro* experiments using two endothelial cell models, HUVECs and TeloHAECs, were carried out to investigate the consequence of high glucose culture conditions on YAP signaling. As shown in Figure 1A, phospho-YAP was decreased in HUVECs exposed to high glucose, as compared to normal glucose. By contrast, no differences were observed on total YAP, phospho-TAZ and total TAZ (Figures 1A,B). In addition, Western blot and qRT-PCR analysis showed that the expression of both CTGF and CYR61 (two transcriptional target genes of YAP/TAZ/TEAD) (Zhao et al., 2008; Shome et al., 2020) were increased by high glucose conditions (Figure 1C and Supplementary Figure 2, respectively). Accordingly, the signal response was increased by high glucose in TeloHAECs transduced with TEAD-luciferase reporter (Figure 2A). The specificity of the model was previously validated by using inactive TEAD-mutant or inhibition with TEAD inhibitor K-975 (Supplementary Figure 3). Taken together, these results suggest that high glucose activates YAP but not TAZ in endothelial cells.

### YAP Is Involved in High Glucose-Induced Endothelial Activation

The consequences of YAP activation in endothelial cells in response to high glucose were characterized in HUVECs by hampering YAP activity by using either siRNA-mediated YAP/TAZ deletion or the TEAD inhibitor K-975. VCAM-1 expression was monitored as a marker of endothelial activation known to be highly sensitive to YAP/TAZ deletion (Choi et al., 2018) and CTGF was chosen as a downstream signal of TEAD activity (Zhao et al., 2008). The expression of VCAM-1 and ICAM-1 was induced by high glucose as assessed by Western blot (Figures 2B,C,E for VCAM-1 and Figure 2E for ICAM-1) and flow cytometry (Figure 2F). siRNA-mediated deletion of YAP/TAZ prevented the increase in VCAM-1 (Figures 2B,C) and CTGF expression (Figures 2B,D) as observed under high glucose exposure. Accordingly, K-975, as a blocker of the YAP/TEAD activation cascade, blunted glucose-mediated alteration of VCAM-1 and ICAM-1 expression and cell surface expression (Figures 2E,F). These findings suggest that YAP/TAZ signaling plays a critical role in high glucose-induced endothelial activation. Confirmation of siRNA-mediated deletion of YAP/TAZ is shown in Supplementary Figure 4. The residual expression of TAZ observed after the siRNA approach is in line with what has been already published by others (Yu et al., 2012; Rausch et al., 2019). Importantly, combination of YAP and TAZ siRNAs was preferred to siRNA YAP alone to efficiently block the pathway and avoid any possible compensation due to the residual expression of TAZ, as already shown (Nallet-Staub et al., 2014; Choi et al., 2018). Supplementary Figure 5 shows that K-975-mediated inhibition of YAP/TEAD prevented the increased expression of the YAP/TEAD downstream effectors CTGF and CYR61 otherwise observed in HUVECs under high glucose, thus confirming the inhibitory effect of K-975 on YAP/TEAD signaling.

### Effects of High Glucose and Shear Stress on YAP Activation in Endothelial Cells

**FIGURE 3:**
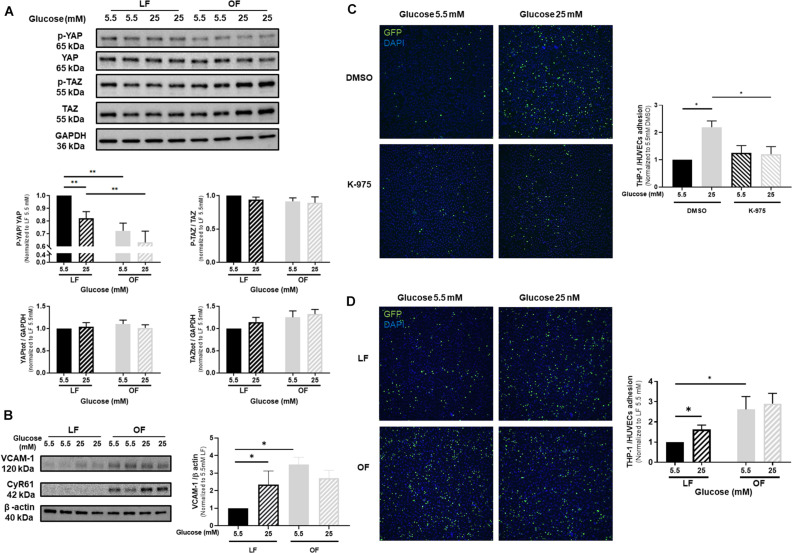
Effects of high glucose and shear stress on YAP activation in endothelial cells. **(A)** Representative protein expression blots and bar graphs of phospho and total YAP, phospho and total TAZ in HUVECs cultured under either normal or high glucose and subjected to either laminar flow (LF, 12 dyn/cm^2^) or oscillatory flow (OF, 0.5 ± 6 dyn/cm^2^; 1 Hz) for 72 h. **(B)** Representative protein expression blots of VCAM-1 and CYR61 and bar graphs of VCAM-1 in HUVECs cultured under either normal or high glucose and subjected to LF or OF for 72 h. **(C)** Representative microphotographs and bar graphs showing THP-1 monocyte attachments to HUVECs cultured under either normal or high glucose, subjected to LF for 72 h and additionally treated with the YAP/TEAD inhibitor K-975 (200 nM). DMSO was used as control for K-975. **(D)** Representative microphotographs and bar graphs showing THP-1 monocyte attachments to HUVECs cultured under either normal or high glucose and subjected to either LF or OF for 72 h. THP-1 monocytes are GFP positive (green fluorescence). Nuclei are stained by DAPI (blue fluorescence). Data are presented as the mean ± SEM. *N* = 4–11/group for panel A; *N* = 6–15/group for panel B; *N* = 4–5/group for panel C and *N* = 5–6/group for panel D. ^∗^*p* < 0.05 and ^∗∗^*p* < 0.01 between the indicated groups. Two-tailed Mann-Whitney test **(A,C,D)** or two-tailed Student’s *t* test **(B)** was used for comparisons between the groups.

Since vascular mechanotransduction was shown to activate YAP/TAZ signaling, we next evaluated the effects of high glucose on YAP phosphorylation in HUVECs exposed to oscillatory flow, compared to laminar flow conditions. Whereas the expression of total YAP, phospho-TAZ and total TAZ was not altered by high glucose under either laminar or oscillatory flow, phospho-YAP significantly declined under laminar flow and high glucose exposure (Figure 3A). YAP phosphorylation was reduced by oscillatory flow conditions, but high glucose additional effect was not significant (Figure 3A). The expression of VCAM-1 (Figure 3B) was increased by high glucose under laminar flow, as compared to normal glucose conditions. Similarly, VCAM-1 expression increased under oscillatory flow conditions, but high glucose in combination with oscillatory flow conditions did not exert any additional effects on VCAM-1 expression (Figure 3B). Interestingly, a similar response was also observed for the expression of CYR61 (Figure 3B and Supplementary Figure 6). Subsequently, the rate of monocyte-endothelial cells attachment was increased by high glucose under laminar flow, as compared to normal glucose conditions (Figure 3C). Conversely K-975 blunted the glucose-induced THP1 monocyte attachments to HUVECs (Figure 3C). The rate of monocyte attachment to HUVECs subjected to oscillatory flow was significantly increased compared to laminar flow exposure (Figure 3D). However, no additional effects were observed when cells were cultured in high glucose conditions (Figure 3D).

### Diabetes Induces YAP Activation in the Vascular Wall of Mice

**FIGURE 4:**
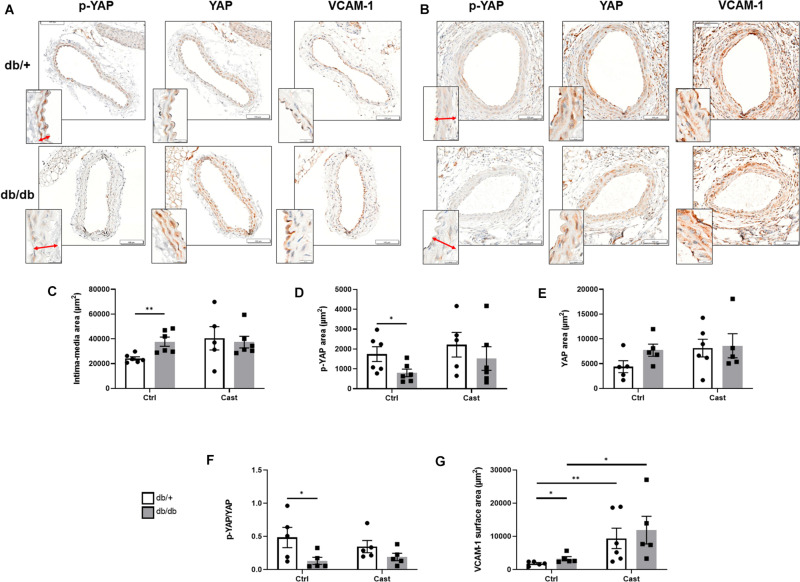
YAP is activated in the vascular wall of diabetic mice. Representative microphotographs (×40 magnification) showing cross sections of either carotid arteries without cast **(A)** or cast-instrumented carotid arteries **(B)** isolated from *db/* + or *db/db* mice and stained for p-YAP, total YAP and VCAM-1. Inserts at the lower left side show a detail of the arterial wall at higher magnification (×100), with the intima-media thickness indicated by an arrow. Bar graphs show the quantification of the intima-media area **(C)**, and the expression of p-YAP **(D)**, total YAP **(E)**, the ratio p-YAP/YAP **(F)** and VCAM-1 **(G)**. Data are presented as the mean ± SEM. *N* = 5–6/group. ^∗^*p* < 0.05, ^∗∗^*p* < 0.01 between the indicated groups. Kruskal-Wallis test was used for comparisons.

Next, we aimed to evaluate the effects of high glucose and shear stress on YAP in the vasculature of type 2 diabetic mice. First, high blood glucose levels in *db/db* mice (658 ± 115 mg/dL) compared to *db/* + mice (255 ± 22 mg/dL) were confirmed before tissue collection. Then, we analyzed the vascular wall of carotid arteries without casts in *db/db* and *db/* + mice. As shown in Figures 4A,C, compared to *db/* + mice, an enlargement of the intima-media thickness was observed in control carotid arteries of *db/db* mice. Phospho-YAP was detected in the vascular wall of *db/* + mice and significantly decreased in *db/db* mice (Figures 4A,D). No difference in the expression of total YAP was observed (Figures 4A,E), thus resulting in a decrease in phospho/total YAP ratio and YAP activation in the vascular wall of diabetic mice (Figure 4F). Additional immunohistochemical analysis showed that the expression of VCAM-1 increased in the carotid artery vascular wall of diabetic mice (Figures 4A,G). Finally, with the exception of VCAM-1 expression, no differences were observed for all other parameters in cast-instrumented carotid arteries compared to carotid arteries without cast (Figures 4B–G), and no differences were observed in cast-instrumented carotid arteries between diabetic and non-diabetic mice (Figures 4A–G).

## Discussion

Previous reports have shown that YAP/TAZ plays a role in initiating and potentiating early steps of atherogenesis (Wang et al., 2012). Understanding the mechanisms that regulate the early steps of atherosclerosis in the settings of diabetes is essential to identify biomarkers and/or therapeutic targets for the treatment of diabetes-associated vascular complications.

First, we observed that only YAP is activated by high glucose and this likely participates in the described diabetes-induced endothelial activation and inflammation (Chen and Harris, 2016). Using two different inhibitory approaches we demonstrated that YAP inhibition reduces the expression of VCAM-1 and prevents monocyte attachment to endothelial cells under high glucose and either static condition or laminar shear stress, thus attenuating high glucose-mediated impairment of endothelial inflammation. However, we do not know whether YAP inhibition could also improve endothelial inflammation in our animal model of type 2 diabetes. Importantly, the effect of high glucose on YAP phosphorylation and VCAM-1 expression observed under laminar flow *in vitro* is consistent with that observed in the vasculature of *db/db* mice without cast. Nonetheless, although a trend was observed between *db*/ + mice with or without cast, the response induced by *in vitro* oscillatory flow on YAP phosphorylation was not observed in the vasculature of mice subjected to carotid cast-induced turbulent flow for 4 weeks. However, we cannot exclude that turbulent flow induced by the cast for shorter or longer time could produce similar effects as those observed *in vitro*. In addition, oscillatory flow imposed *in vitro* on endothelial cells might not fully be translatable in nature and magnitude with the turbulent flow generated downstream of the cast. Vascular regions exposed to disturbed flow are known to be more prone to atherogenesis (Cunningham and Gotlieb, 2005; Wang Y. et al., 2016). On the other side, vascular regions exposed to laminar flow are protected against endothelial inflammation and atherogenesis (Chen et al., 2003; Xu et al., 2016). Interestingly, our *in vitro* and *in vivo* observations suggest that high glucose exerts a detrimental effect (increased inflammation) on vascular regions exposed to laminar flow, which is likely due to YAP activation and that may confer these vascular regions an atheroprone phenotype similar to that observed in vascular regions exposed to oscillatory flow. In our study, we found that the combination of high glucose with oscillatory flow does not further decrease YAP phosphorylation (although a trend was observed both *in vitro* and *in vivo*), and this could be due to the fact that YAP is already maximally activated (dephosphorylated) by either high glucose or oscillatory flow alone. Indeed, although from different origin, high glucose on vascular regions exposed to laminar flow and oscillatory flow seems to exert similar effects by converging to the YAP signaling pathway. Importantly, our study focused on the effect of high glucose and shear stress on YAP activation in endothelial cells. However, we cannot exclude that high glucose and/or shear stress may also affect YAP activation in other vascular cells (like vascular smooth muscle cells), and further studies are needed to clarify these aspects.

To date, the involvement of YAP/TAZ signaling in the context of diabetes has only been shown in pathologies that are not directly associated with vascular diseases. The expression of YAP and CTGF increase *in vitro* and *in vivo* in renal proximal tubule epithelial cells under high glucose and in the kidney of type 2 diabetic patients, indicating that YAP may play a critical role in renal damage associated with diabetes (Chen and Harris, 2016; Ma et al., 2019). Several studies have also shown the crosstalk between YAP activation and high glucose in different types of cancers. In particular, it has been shown that high glucose increases O-GlcNAcylation, which is an important post-translational protein modification that plays pro-oncogenic roles (Teo et al., 2010; Peng et al., 2017; Zhang et al., 2017). More precisely, in breast cancer cells, O-GlcNAcylation of YAP was shown to precede its dephosphorylation (Peng et al., 2017). O-GlcNAcylation could also be the main driver of YAP activation in the diabetic vasculature. Moreover, it has also been demonstrated that mechanical stimuli modulate YAP/TAZ activities through RhoA in the vasculature (Dupont et al., 2011; Wang K. C. et al., 2016). A limitation of our study is that we didn’t evaluate whether glucose induces O-GlcNAcylation which, in turn, may promote YAP activation in endothelial cells or in the diabetic vasculature, and whether this mechanism converges (or not) to the RhoA-mediated mechanism that may induce YAP activation by oscillatory flow. However, to understand the exact mechanisms additional studies using specific knockout animal models should be performed. Additionally, antidiabetic drugs such as metformin and liraglutide, also known to attenuate the development of atherosclerosis, were recently suspected to activate the hippo pathway independently of glucose lowering effect (Li et al., 2018; Wu et al., 2019; Zhang J. J. et al., 2019). Therefore, blocking YAP activation may elicit their beneficial effects observed in the setting of atherosclerosis and vascular dysfunction (Li et al., 2017; Wang et al., 2017).

In conclusion, we demonstrate for the first time that YAP is activated by high glucose and perturbed shear stress in endothelial cells, and that inhibition of YAP attenuates cell activation and monocyte attachment which is otherwise promoted by high glucose. Although translation *in vivo* of YAP inhibition remains to be tested, these findings bring the first evidences that in vascular regions exposed to laminar flow, hyperglycemia may promote an atheroprone phenotype like that occurring in vascular regions exposed to oscillatory flow, and that YAP inhibition may be a potential therapeutic opportunity to improve vascular complications associated with diabetes.

## Data Availability Statement

The raw data supporting the conclusions of this article will be made available by the authors, without undue reservation.

## Ethics Statement

The animal study was reviewed and approved by the IACUC of Sanofi R&D.

## Author Contributions

JO, J-CL, EV, BL, CG, and LL performed the *in vitro* experiments. JO, BP, and SB performed the *in vivo* experiments. JO, VB, and PB performed the immunohistochemical staining and analyses. JO, J-CL, EV, SN, PJ, EG, and MM wrote and edited the manuscript. All authors contributed to the article and approved the submitted version.

## Conflict of Interest

All authors are Sanofi employees. The authors declare that the research was conducted in the absence of any commercial or financial relationships that could be construed as a potential conflict of interest.
