# Supplementary material for: High Glucose Activates YAP Signaling to Promote Vascular Inflammation
Source: Front Physiol. 2021 Jun 4;12:665994. doi: 10.3389/fphys.2021.665994 (PMC8213390; doi:10.3389/fphys.2021.665994)
Supplement: Supplementary file 1 [file Data_Sheet_1.docx]

Supplementary Material

# Supplementary Figures

**
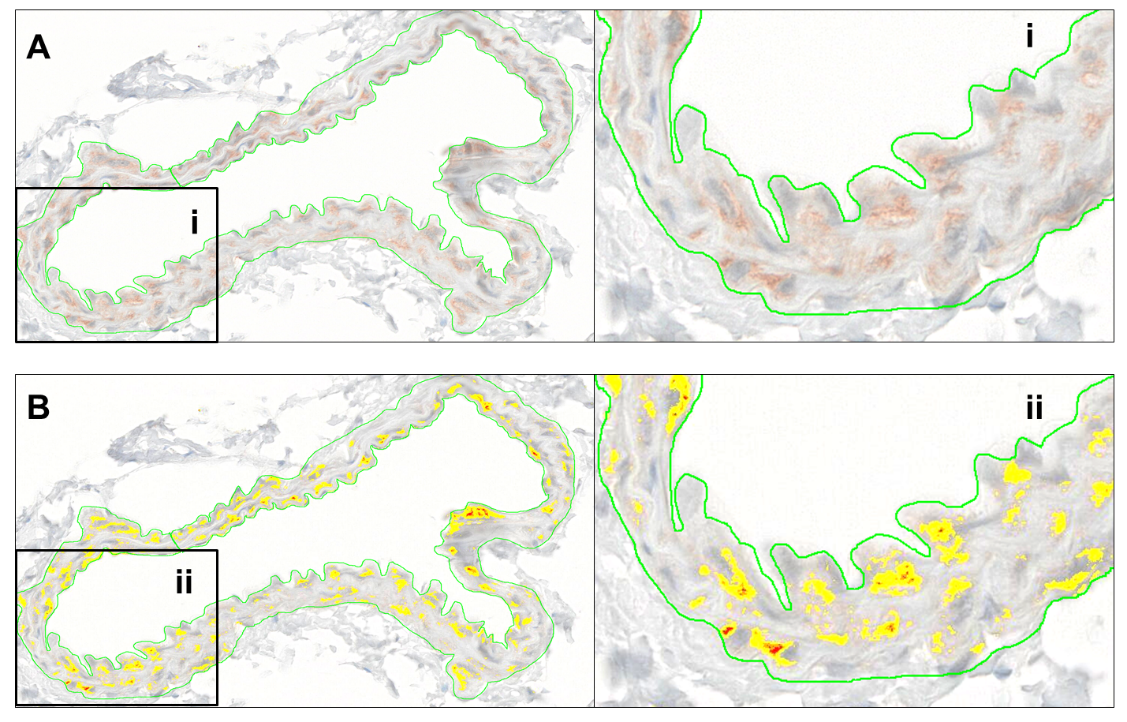
**

**Supplementary Figure 1. Description of immunostaining quantification.** Representative microphotographs showing how quantification of staining intensity was performed. A representative image of a carotid artery after staining for p-YAP is shown here. (A) Representative microphotograph (x40) shows how the intima-media area was delimited (in green). The insert (i) shows the indicated region at higher magnification (x100). (B) Representative microphotograph shows how quantification of staining intensity was performed using the Indica Labs HALO software, which automatically recognizes and quantifies different immunolabelling color intensities including weak (in yellow), moderate (in orange) and strong (in red) positive pixels. The insert (ii) shows details of immunolabelling color intensities at higher magnification.

**
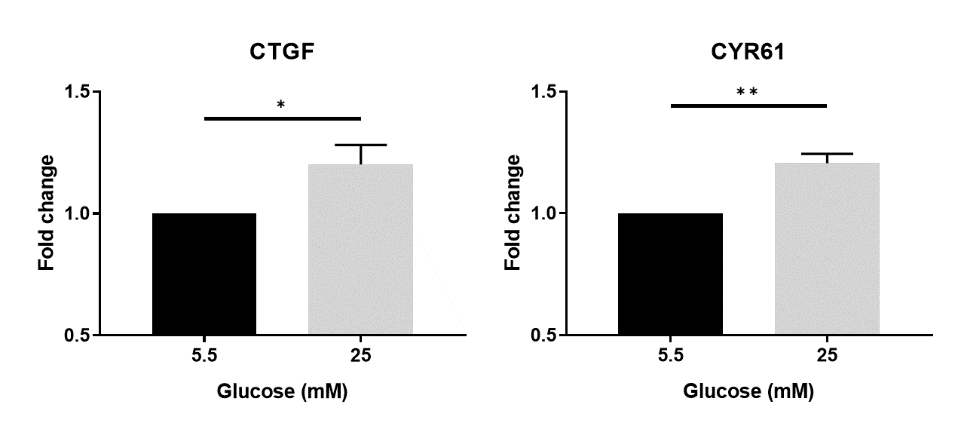
**

**Supplementary Figure 2. Effect of high glucose on YAP/TAZ/TEAD target genes.** qRT-PCR showing the increased expression of CTGF and CYR61 in HUVECs under high glucose conditions. Data are presented as the mean ± SEM. N= 5/group (two-tailed Student`s t test). **p*<0.05 and ***p*<0.01 between the groups.

**
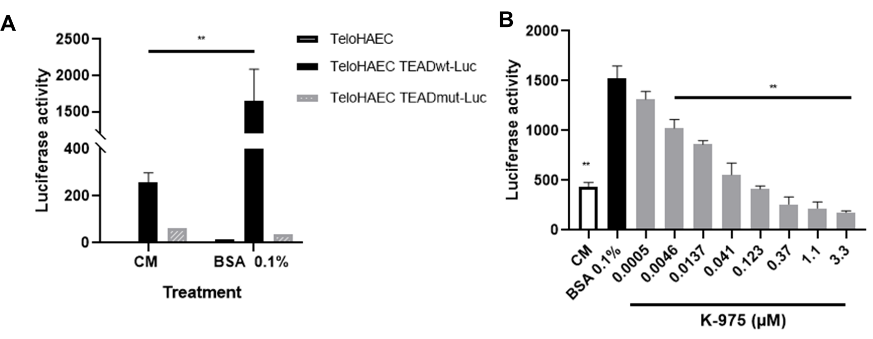
**

**Supplementary Figure 3. Validation of TEAD-luciferase reporter in TeloHAECs.** Bar graphs show the activity of TEAD-luciferase in TeloHAECs. (A) Untransduced or transduced TeloHAECs with wild type or 7x mutated constructs (TeloHAEC, TeloHAEC TEADwt-Luc and TeloHAEC TEADmut-Luc, respectively) were cultured in complete medium (CM) or basal medium supplemented with 0.1% BSA. (B) TeloHAEC TEADwt-Luc cells were maintained in CM or vascular cell basal medium supplemented with 0.1% BSA with or without the YAP/TEAD inhibitor K-975. All media contained glucose 5.5mM. Data are presented as the mean ± SEM. N= 2-6/group (Mann-Whitney test).  **p*<0.05 and ***p*<0.01 versus BSA 0.1 % and ***p*<0.01 between indicated groups.

**
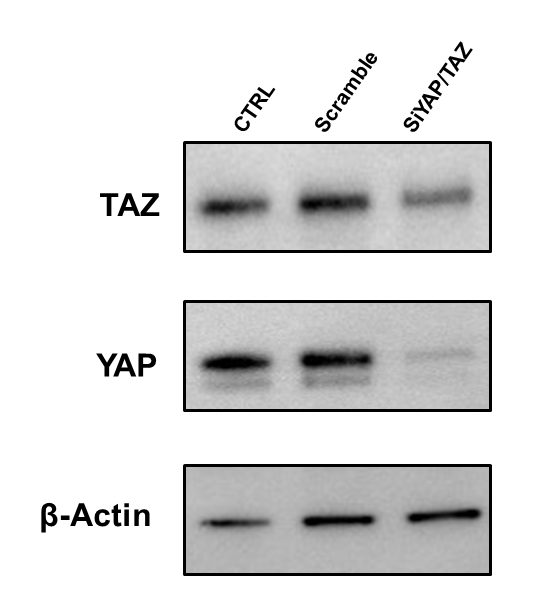
**

**Supplementary Figure 4. Validation of siRNA-mediated deletion of YAP and TAZ.** Representative protein expression blots showing the siRNA-mediated deletion of YAP and TAZ in HUVECs.

**
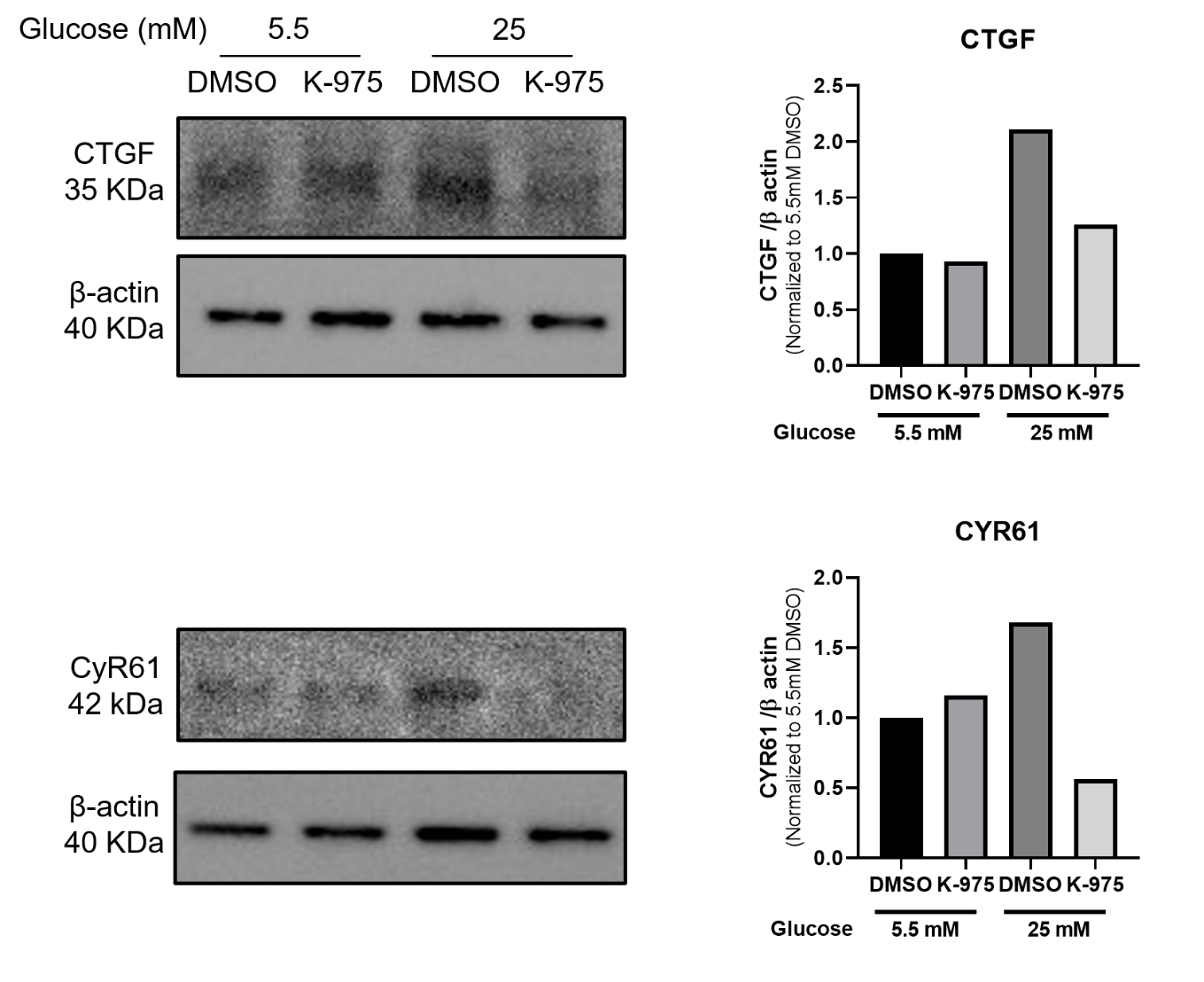
**

**Supplementary Figure 5. Validation of K-975-mediated inhibition of YAP/TAZ signaling.** Representative protein expression blots and bar graphs showing the expression of CTGF and CYR61 in HUVECs cultured under static conditions with either normal or high glucose for 24 hours and in the presence, or not, of the YAP/TEAD inhibitor K-975 at 200nM. DMSO was used as control.

**
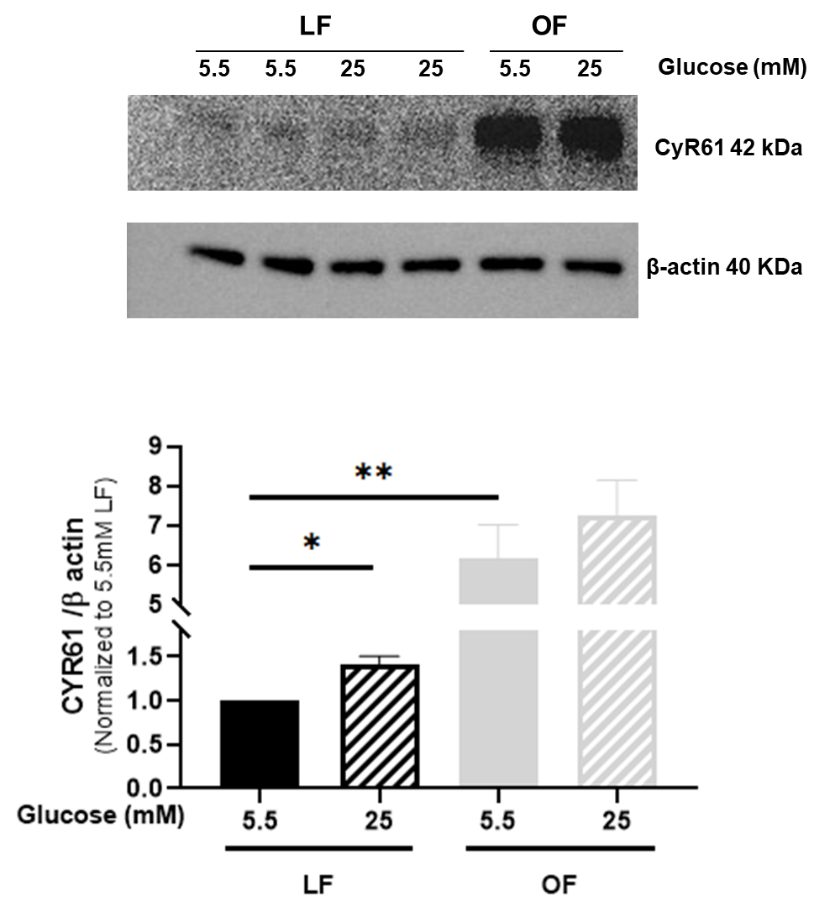
**

**Supplementary Figure 6. Effect of high glucose and shear stress on the YAP/TAZ/TEAD target gene CYR61.** Representative protein expression blots and bar graphs showing the expression of CYR61 in HUVECs cultured under either normal or high glucose and subjected to LF or OF for 72 hours. Data are presented as the mean ± SEM. N= 5-6/group (two-tailed Student`s t test). **p*<0.05 and ***p*<0.01 between the indicated groups.
